# Supplementary material for: Clinical, financial and social impacts of COVID-19 and their associations with mental health for mothers and children experiencing adversity in Australia
Source: PLoS One. 2021 Sep 13;16(9):e0257357. doi: 10.1371/journal.pone.0257357 (PMC8437268; doi:10.1371/journal.pone.0257357)
Supplement: S1 Table — (DOCX) [file pone.0257357.s001.docx]

**S1 Table. Summary data of COVID-19 impacts (individual items and summary measures) and mental health outcomes, broken down by State and date of assessment.**

|  | By State | | | By Date | | |
| --- | --- | --- | --- | --- | --- | --- |
|  | Vic  n=210 | Tas  n=97 | Other  n=12 | 6 May-1 Jun  n=71 | 2 Jun-7 Jul  n=128 | 8 Jul-6 Dec  n=120 |
| **Clinical exposure** |  |  |  |  |  |  |
| Mother – positive test result | 1 (0.5) | 0 (0.0) | 0 (0.0) | 0 (0.0) | 1 (0.8) | 0 (0.0) |
| Mother – negative test result | 32 (15.3) | 5 (5.2) | 0 (0.0) | 8 (11.3) | 16 (12.5) | 13 (10.9) |
| Mother self-quarantined | 44 (21.1) | 12 (12.4) | 0 (0.0) | 12 (16.9) | 22 (17.2) | 22 (18.5) |
| Child – positive test result | 1 (0.5) | 0 (0.0) | 0 (0.0) | 0 (0.0) | 1 (0.8) | 0 (0.0) |
| Child – negative test result | 17 (8.2) | 4 (4.3) | 0 (0.0) | 0 (0.0) | 6 (4.7) | 15 (12.9) |
| Child self-quarantined | 29 (13.9) | 7 (7.2) | 0 (0.0) | 5 (7.0) | 13 (10.2) | 18 (15.1) |
| Household family – positive test result | 13 (6.2) | 8 (8.3) | 2 (16.7) | 2 (2.8) | 8 (6.3) | 13 (10.9) |
| Non-household family – positive test result | 7 (3.4) | 1 (1.0) | 0 (0.0) | 0 (0.0) | 6 (4.7) | 2 (1.7) |
| *Mother/child self-quarantine summary* | *49 (23.4)* | *14 (14.4)* | *0 (0.0)* | *12 (16.9)* | *25 (19.5)* | *26 (21.9)* |
| **Changes to financial circumstances** |  |  |  |  |  |  |
| Mother reduced ability to earn | 46 (22.0) | 16 (16.5) | 3 (25.0) | 16 (22.5) | 27 (21.1) | 22 (18.5) |
| Mother lost job | 12 (5.7) | 9 (9.3) | 1 (8.3) | 8 (11.3) | 8 (6.3) | 6 (5.0) |
| Family member reduced ability to earn | 35 (16.8) | 15 (15.5) | 1 (8.3) | 17 (23.9) | 20 (15.6) | 14 (11.8) |
| Family member lost job | 26 (12.4) | 9 (9.3) | 1 (8.3) | 12 (16.9) | 11 (8.6) | 13 (10.9) |
| *Mother lost job/income summary* | *56 (26.8)* | *25 (25.8)* | *4 (33.3)* | *24 (33.8)* | *35 (27.3)* | *26 (21.9)* |
| **Current financial hardship** |  |  |  |  |  |  |
| Financial difficulties – mortgage, rent, loan repayments | 13 (6.2) | 8 (8.3) | 1 (8.3) | 4 (5.6) | 9 (7.0) | 9 (7.6) |
| Financial difficulties – household bills | 32 (15.3) | 11 (11.3) | 1 (8.3) | 10 (14.1) | 21 (16.4) | 13 (10.9) |
| Financial difficulties – food | 14 (6.7) | 4 (4.1) | 1 (8.3) | 4 (5.6) | 6 (4.7) | 9 (7.6) |
| Financial difficulties – healthcare | 7 (3.4) | 2 (2.1) | 1 (8.3) | 1 (1.4) | 7 (5.5) | 2 (1.7) |
| Financial difficulties – home, car insurance | 13 (6.2) | 5 (5.2) | 2 (16.7) | 5 (7.0) | 5 (3.9) | 10 (8.4) |
| *Total financial hardship summary* (mean, (SD) [range]) | *0.4 (0.9)* | *0.3 (0.8)* | *0.5 (1.4)* | *0.3 (0.8)* | *0.4 (0.8)* | *0.4 (1.0)* |
| **Family stress and resilience** |  |  |  |  |  |  |
| *Family stress summary* (mean, (SD) [range]) | *2.2 (0.7)* | *1.9 (0.7)* | *2.1 (0.5)* | *2.2 (0.8)* | *2.2 (0.7)* | *2.0 (0.7)* |
| *Family resilience summary* (mean, (SD) [range]) | *3.3 (0.6)* | *3.3 (0.6)* | *3.2 (0.4)* | *3.3 (0.6)* | *3.3 (0.5)* | *3.2 (0.6)* |
| **Mental health outcomes** |  |  |  |  |  |  |
| Maternal mental health (mean, (SD) [range]) | 9.0 (8.8) | 11.2 (10.9) | 6.3 (7.2) | 9.9 (9.8) | 9.0 (8.1) | 10.0 (10.8) |
| Child mental health (mean, (SD) [range]) | 2.3 (0.7) | 2.3 (0.7) | 2.0 (0.5) | 2.4 (0.8) | 2.4 (0.7) | 2.1 (0.6) |
