# Supplementary material for: Clinical, financial and social impacts of COVID-19 and their associations with mental health for mothers and children experiencing adversity in Australia
Source: PLoS One. 2021 Sep 13;16(9):e0257357. doi: 10.1371/journal.pone.0257357 (PMC8437268; doi:10.1371/journal.pone.0257357)
Supplement: Attachment — Submitted filename: R1 Plos_Response to reviewer comments.docx [file pone.0257357.s002.docx]

| **Editorial office requirements** | **Author Response** | **Location of change** |
| --- | --- | --- |
| 1. Please ensure that your manuscript meets PLOS ONE's style requirements, including those for file naming. | We have reviewed all formatting requirements and updated the manuscript and files accordingly, including adding supporting information captions, adding line numbers and revising the formatting of the title and author page. We have also updated file names as needed. |  |
| 2. In ethics statement in the manuscript and in the online submission form, please provide additional information about the ethics approval for this study. Please clarify whether the initial ethics approval encompass this study or if there was specific approval for a COVID-specific study as part of the right@home study. If not, please confirm with your IRB that specific, prospective approval for the current study was not necessary and attach the correspondence as an 'Other' file for our records ('Other' files are not published). Thank you for your attention to this request. | The ethics approval provided for the larger right@home trial encompasses the COVID-19 follow-up study presented in the manuscript. We have amended the ethics statement in the manuscript, which now states:  The right@home trial, *including the COVID-19 follow-up, were* approved by the Royal Children’s Hospital Human Research Ethics Committee (HREC 32296), Australia.  This clarification has also been made in the online submission form. |  |
| 3. We note that you have indicated that data from this study are available upon request. PLOS only allows data to be available upon request if there are legal or ethical restrictions on sharing data publicly. For information on unacceptable data access restrictions, please see <http://journals.plos.org/plosone/s/data-availability#loc-unacceptable-data-access-restrictions>.   In your revised cover letter, please address the following prompts:  a) If there are ethical or legal restrictions on sharing a de-identified data set, please explain them in detail (e.g., data contain potentially identifying or sensitive patient information) and who has imposed them (e.g., an ethics committee). Please also provide contact information for a data access committee, ethics committee, or other institutional body to which data requests may be sent.  b) If there are no restrictions, please upload the minimal anonymized data set necessary to replicate your study findings as either Supporting Information files or to a stable, public repository and provide us with the relevant URLs, DOIs, or accession numbers. Please see http://www.bmj.com/content/340/bmj.c181.long for guidelines on how to de-identify and prepare clinical data for publication. For a list of acceptable repositories, please see <http://journals.plos.org/plosone/s/data-availability#loc-recommended-repositories>.  We will update your Data Availability statement on your behalf to reflect the information you provide. | We have stated that data are available upon request, as the data reported in this study contain sensitive participant information which are restricted to use for research purposes only and therefore cannot be made publicly available. This restriction is in accordance with our Participant Information and Consent Form and approved study protocol, governed by the Royal Children’s Hospital Human Research Ethics Committee (HREC 32296). We have responded with this information in our cover letter and updated our Data Availability statement accordingly in the online submission. In addition, we include details as to how researchers can request access to the data via the Melbourne Children’s Campus data access platform LifeCourse or the Royal Children’s Hospital Human Research Ethics Committee.  Updated data statement:  Data cannot be shared publicly because they contain sensitive participant information which are restricted to use for research purposes only. This restriction is in accordance with the Participant Information and Consent Form and approved study protocol, governed by the Royal Children’s Hospital Human Research Ethics Committee (HREC 32296). We invite researchers to request access to the data from the Melbourne Children's Campus LifeCourse institutional data access platform (https://lifecourse.melbournechildrens.com/data-access/) or the governing Royal Children’s Hospital HREC (https://www.rch.org.au/ethics/). |  |
| 4. Please include captions for your Supporting Information files at the end of your manuscript, and update any in-text citations to match accordingly. Please see our Supporting Information guidelines for more information: http://journals.plos.org/plosone/s/supporting-information. | We have added the Supporting Information captions to the manuscript and updated in-text citations. |  |
| 5. Please review your reference list to ensure that it is complete and correct. If you have cited papers that have been retracted, please include the rationale for doing so in the manuscript text, or remove these references and replace them with relevant current references. Any changes to the reference list should be mentioned in the rebuttal letter that accompanies your revised manuscript. If you need to cite a retracted article, indicate the article’s retracted status in the References list and also include a citation and full reference for the retraction notice. | All references have been checked that they are complete and correct. Minor changes were made where updated publication details were available and two additional citations were included as part of the response to reviewer comments. |  |
| **Reviewer 1** |  |  |
| 6. The authors didn't make their data public: this might be in violation of PlosOne policies. The authors declare that this is due to data containing potentially re-identifiable and sensitive participant information. Given the peculiarities of the sample, this might be the case, and the authors explained very well the procedure to request access to the data. | We have further clarified the restrictions that mean we are unable to make these data publicly available, in line with the PLOS ONE policies. The data reported in this study contain sensitive participant information which are restricted to use for research purposes only and therefore cannot be made publicly available. This restriction is in accordance with our Participant Information and Consent Form and approved study protocol, governed by the Royal Children’s Hospital Human Research Ethics Committee (HREC 32296). We have updated our Data Availability statement accordingly:  Data cannot be shared publicly because they contain sensitive participant information which are restricted to use for research purposes only. This restriction is in accordance with the Participant Information and Consent Form and approved study protocol, governed by the Royal Children’s Hospital Human Research Ethics Committee (HREC 32296). We invite researchers to request access to the data from the Melbourne Children's Campus LifeCourse institutional data access platform (https://lifecourse.melbournechildrens.com/data-access/) or the governing Royal Children’s Hospital HREC (https://www.rch.org.au/ethics/).  We note that the reviewer is satisfied with our process for accessing data, which can be requested from the Melbourne Children's Campus Lifecourse institutional data platform (https://lifecourse.melbournechildrens.com/data-access/). |  |
| 7. Regarding the introduction, I don't have any particular comment, as it is well written and coherent. I have, however, some troubles with the pictures, as in the submitted files their quality is too poor. Fig1, in particular, is impossible to read, and the other figures are of very degraded quality. This needs to be amended prior to publication. | Thank you for your comments on the introduction. With regard to the images, these have been formatted to meet the PLOS submission requirements. The versions we have uploaded are of high quality and are readable. The reviewer may have found that the images displayed in the pdf proof are of poor quality, but these can be viewed in full quality when downloaded as an image file. If this remains an issue, we can review further at the request of the editorial office. |  |
| 8. Regarding the methods, the study seems to be carefully designed and the methods are adequately described. There is a limitation regarding the sample, as this is a convenience sample recruited from a trial in 2013/14 with several inclusion criteria: this clearly impacts the generalizabilty of the results. The authors only partially address this in the discussion (p21 line unknown as line numbering disappeared from page 18...): I think the authors should state not only the limit itself, but also the potential impact it has. | We have added further discussion of the origin of this cohort as a sample recruited for a randomised controlled trial of nurse home visiting. Given that these are families that have remained engaged in a longitudinal research trial over a sustained period, they are likely to under-represent the impacts of the pandemic for those experiencing more acute levels of adversity. We have added:  *Through the recruitment and retention processes of the trial, we have retained a cohort with experiences of adversity that are traditionally not well represented in longitudinal studies.[45]*  And:  *The study includes a cohort of participants who were already engaged in a randomised trial of nurse home visiting and who had met the initial screening and inclusion criteria of the trial. Given that the families in this cohort have remained engaged in a longitudinal research project for a sustained period of 5 to 6 years, it is likely that the findings under-represent some of the families who were most socially vulnerable prior to and over the course of the pandemic. Hence the findings we describe may understate the full impact of the pandemic on families we couldn’t represent, such as* *mothers without literacy in English who did not meet the eligibility criteria of the trial or those experiencing high levels of adversity who were lost to follow-up over the course of the trial.* | Page 26, line 435  Page 26, line 447-456 |
| 9. Study measures are well described, and Table 1 is quite useful. However, may I suggest the authors to add in the supplementaries a copy of the survey? This would help replicability, and potentally allow the authors to remove some details from the text. | We have added details to the references for both the DASS-21 and the CRISIS 3.0 measures, to include the direct link to the measures, which are freely accessible online. We have retained the measure details in Table 1 as it provides information about how items were scored and analysed for interpretation of the results. | References [36] and [39] |
| 10. Maternal mental health and child mental health were assessed on two different time-frames (respectively, past week and past 2 weeks). Why? | Existing, validated measures of maternal and child mental health were used, which both have pre-specified periods of assessment. We recognise that it would be beneficial to have the timeframes of these measures align; however, we prioritised using the validated measures as designed and ensuring comparability within and between studies. We note that the difference in timeframes is small.  We have added the following clarifications to the description of the measures:  *Maternal mental health was measured using the 21-item short form of the Depression Anxiety and Stress Scales (DASS-21).[39] The DASS-21 was selected as it is a widely used and validated measure of mental health [40] and is consistent with the repeated measurement throughout the right@home trial.*  *Child mental health and wellbeing was measured using the 8-item parent-reported CoRonavIruS Health Impact Survey 3.0 Child Mood States scale (CRISIS 3.0).[35, 36] The CRISIS 3.0 Child Mood States scale was selected as a newly validated but widely adopted measure of child mental health in the context of the COVID-19 pandemic.* | Page 13, line 216  Page 13, line 225 |
| 11. Statistical analyses were performed, to the best of my knowledge, adequately. However, I don't understand why the authors decided to carry out and report 3 different models. What are model 1 and 2 adding to the results? If you hypothesise that all the measures of COVID-19 impact are contributing in determining you dependent variables, then only model 3 is relevant. Otherwise, the reason for this should be carefully explained in the manuscript. | We present the three models as each provides important information about the associations between the COVID-19 impacts and mental health outcomes. As there is likely co-occurrence between each of the COVID-19 impacts as well as pre-COVID maternal mental health it is not feasible to fully delineate the specific effects of each of these. We therefore provide each of the models, to allow readers to interpret the associations in the context of each level of adjustment. We have added detail to the methods (changes underlined):  *Linear regression models examined COVID-19 impacts for associations with maternal and child mental health. Exposures (COVID-19 impacts) were first examined individually to identify evidence of associations for each COVID-19 impact with maternal and child mental health, adjusting for pre-COVID-19 characteristics of household income, mother’s marital status and mother’s education; State of residence at the time of COVID-19 data collection (Vic, Tas or Other); and randomisation status (intervention versus control) (Model 1). A second series of models additionally adjusted for pre-COVID-19 maternal mental health (Model 2). Finally, all exposures (COVID-19 impacts) were analysed in a single model to identify evidence of their unique associations with maternal and child mental health, after accounting for each of the other exposures. Model 3 also adjusted for all potential covariates included in Model 2. We anticipated that there would be co-occurrence between each of the COVID-19 impacts and pre-COVID-19 maternal mental health; we therefore present all models to allow interpretation of these associations at each level of adjustment.* | Page 14, line 260-272 |
| 12. Regarding the regression models, the authors should also report the coefficient of determination (R2) for each model | As suggested, we have added the R^2^ in Table 4 for all models. We have also made the following changes to the text in the Results (changes underlined):  *Regression coefficients (β) are presented as standardised coefficients, which can be interpreted as the number of standard deviations difference in the mental health outcome (comparable to effect sizes). The R-squared statistic (R2) is also shown as the proportion of the variance in maternal and child mental health that is explained by each model. When examining each COVID-19 impact as individual exposures in separate regression models, any self-quarantine (β=0.46, p=0.003), greater financial hardship (β=0.27, p<0.001) and more family stress (β=0.49, p<0.001) were all associated with higher self-reported maternal mental health symptoms, while greater family resilience was associated with lower maternal mental health symptoms (β=-0.40, p<0.001). These associations each accounted for pre-COVID-19 characteristics, state of residence and randomisation status (Model 1). The variance explained by each of these models ranged from 5% (any self-quarantine) to 13% (family stress). Associations for self-quarantine, greater financial hardship and family stress were also evident after additionally controlling for pre-COVID-19 maternal mental health, while the association for family resilience was attenuated (Model 2). Similar associations were identified for child mental health, although maternal job or income loss was associated with poorer child mental health (β=0.37, p=0.006) while overall financial hardship was not (Model 1). The variance explained by each of these models ranged from 7% (maternal job or income loss) to 31% (family stress). The associations for child mental health were all still evident after additionally controlling for pre-COVID-19 maternal mental health (Model 2).*  *When all COVID-19 impacts were examined in a single model (Model 3), accounting for any co-occurring effects of the exposures, greater family stress showed the strongest association with both maternal (β=0.31, p<0.001) and child (β=0.68, p<0.001) mental health, and greater family resilience was associated with lower child mental health symptoms (β=-0.36, p<0.001). Other associations were attenuated slightly. The variance explained by the models when accounting for all COVID-19 impacts as well as pre-COVID characteristics state of residence, randomisation status and pre-COVID-19 maternal mental health was 42% for maternal mental health and 39% for child mental health.* | Page 19, line 326-345  Page 23, line 356-363 |
| 13. In table 4 the cross near Model 1 has no footnote | The † represents the footnote for Model 1. We have clarified in the footnotes which models they refer to for clarity. The footnote now reads:  † *Model 1 was adjusted for pre-COVID family income (paid employment vs benefit or pension), marital status (married/living with partner vs not), maternal education (did not vs did complete high school or any further education); state at the time of COVID-19 data collection (Vic, Tas or Other); and randomisation status (intervention vs control).* | Table 4, page 22 |
| 14. In conclusion, this is an overall well written paper, describing a potentially interesting study. There are some issues with the figure quality and some minor changes to be done in the methods/results sections. | We thank the reviewer for their comments. We have addressed their concerns in the items above. |  |
| **Reviewer 2** |  |  |
| 15. I thank you for the opportunity to review this manuscript which is very timely and falls within my area of interest. Analyses are well done and results have been clearly described. I suggest to the authors some changes to improve the quality of their work. In particular I would suggest author to expand - in the introduction section - the literature on the COVID-19 pandemic psychosocial impact on citizens in order to more widely discuss your results. | We thank the reviewer for their comments and noting the timeliness and interest in this research. We have added additional literature detailing the psychosocial impacts of COVID-19 on families. The introduction now includes (additional details underlined):  *Families reporting poorer mental health were also more likely to have lost access to regular childcare and be experiencing financial hardship (assessed as food insecurity). These findings were again mirrored by those of a UK study which surveyed families in a highly deprived and ethnically diverse city in April to June 2020; this study found high rates of poor mental health amongst mothers (39-43%) and that poor mental health was also associated with unemployment, housing insecurity and lack of social support during the public health restrictions.[7]*  *With regard to children’s mental health, an online survey of 5823 parents in Austria, Germany Liechtenstein and Switzerland in April to May 2020 found that 33-43% of parents reported an increase in their child’s emotional and behavioural difficulties from the onset of the pandemic, amongst 1- to 6-year-olds.[31] Similarly, a nationally representative study in Germany which surveyed families between May to June 2020 found that rates of mental health difficulties had approximately doubled from pre-pandemic figures, amongst 7- to 17-year-olds. Again, these increases in poor mental health were greatest amongst families experiencing socioeconomic adversity and were also greater amongst younger children.[32] For families who were experiencing adversity before the pandemic, limited resources and supports are likely to be further diminished by both the financial and social effects of the pandemic and related restrictions.[10] These impacts are expected to increase the risk of poor parent and child mental health amongst families experiencing adversity, with children bearing a disproportionate burden.* | Page 6, line 115-135 |
